# Broad network-based predictability of *Saccharomyces cerevisiae *gene loss-of-function phenotypes

**DOI:** 10.1186/gb-2007-8-12-r258

**Published:** 2007-12-05

**Authors:** Kriston L McGary, Insuk Lee, Edward M Marcotte

**Affiliations:** 1 Center for Systems and Synthetic Biology, Institute for Cellular and Molecular Biology, University of Texas at Austin, 2500 Speedway, Austin, Texas 78712, USA; 2 Department of Chemistry & Biochemistry, University of Texas at Austin, 2500 Speedway, Austin, Texas 78712, USA

## Abstract

We demonstrate that loss-of-function yeast phenotypes are predictable by guilt-by-association in functional gene networks. Testing 1,102 loss-of-function phenotypes from genome-wide assays of yeast reveals predictability of diverse phenotypes, spanning cellular morphology, growth, metabolism, and quantitative cell shape features. We apply the method to extend a genome-wide screen by predicting, then verifying, genes whose disruption elongates yeast cells, and to predict human disease genes. To facilitate network-guided screens, a web server is available .

## Abstract 2

Loss-of-function phenotypes of yeast genes can be predicted from the loss-of-function phenotypes of their neighbours in functional gene networks. This could potentially be applied to the prediction of human disease genes.

## Background

Geneticists have long observed that mutations that lead to the same organismal phenotype are typically functionally related, and have interpreted epistatic relationships between genes as genetic pathways and more recently as gene networks. In the post-genomic period, an abundance of high-throughput data has encouraged the construction of functional networks [1], which integrate evidence from a wide variety of experiments to infer functional relationships between genes. Historically, mutations that lead to the same phenotype were inferred to be functionally linked; now, with extensive functional networks, we ask whether the inverse is also true. If gene loss-of-function phenotypes could be successfully inferred on the basis of linkages in functional gene networks, then this would enable the directed extension of genetic screens and open the possibility to apply similar approaches in humans for the direct identification of disease genes.

In particular, important advances over the past decade in both forward and reverse genetics mean that such predictability could be exploited in a straightforward manner to associate specific genes with phenotypes. In terms of forward genetics, genome-wide association studies (for review, see [2]) are showing great power for identifying candidate genes associated with human traits and diseases, such as recent studies correlating variants in the *ORMDL3 *gene with risk for childhood asthma [3]. In terms of reverse genetics, rapid testing of candidate genes has become more routine because of availability of mutant strain collections (for example, yeast deletion strain collections [4,5]) as well as the relative ease of RNA interference downregulation of genes (as, for instance, for genome-wide RNA interference screens of *Caenorhabtidis elegans *[6,7] or human cell lines; for review [8]). The prediction of loss-of-function phenotypes would bridge these two aspects of genetics; given an initial set of genes associated with a phenotype of interest, such as might come from either forward or reverse genetics, computational predictions of additional genes associated with that phenotype might be rapidly tested using reverse genetics, thereby extending the original screen. Most importantly, because many traits are multifactorial in nature, often based upon contributions from many genes, such approaches might help in defining networks of genes that affect a trait of interest. The potential for discovering such polygenic contributions to traits appears to be particularly strong when one considers the prediction of phenotypes directly from functional gene networks.

Functional linkages - statistical associations between pairs of genes that are likely to participate in the same cellular pathway or process - have shown great general power for generating hypotheses about gene function, in spite of their apparently nonmechanistic nature (for examples, see [9-18]). In a probabilistic functional gene network, each linkage in the network is scored with the likelihood of the linked genes belonging to the same pathway [13,16,17]. The accuracy and coverage of these networks depends on the integration of multiple data sources (protein interactions, DNA microarrays, literature mining, and so on) that have each been independently shown to link similarly annotated genes; the combination of many such datasets means that the networks often extend well beyond current annotation. Such networks have therefore been extensively applied to infer gene function, such as by predicting an uncharacterized gene's function on the basis of its network neighbors (for examples, see [9,13,15,19-22]). Because genes linked in these networks tend to be in the same pathway, it is reasonable also to expect linked genes to often share loss-of-function phenotypes.

In this report we show proof-of-principle that genes linked in a functional network are indeed likely to give rise to the same loss-of-function phenotype, demonstrating efficacy for predicting yeast mutant phenotypes. Diverse yeast gene loss-of-function phenotypes are shown to be predictable, from biochemical to morphologic to fitness effects. The approach we describe therefore provides a rational and quantitative foundation for targeted reverse genetic studies, as we demonstrate by predicting, then verifying, essential genes whose disruption produces elongated yeast cells. The breadth of applicability suggests that this approach might ultimately be valuable if it is implemented in humans to identify genes that are likely to lead to human disease, exploiting extensive functional genomics data and sets of known disease genes in order to identify directly new candidate disease genes.

## Results

### Guilt-by-association in a functional gene network predicts yeast gene essentiality

**Figure 1:**
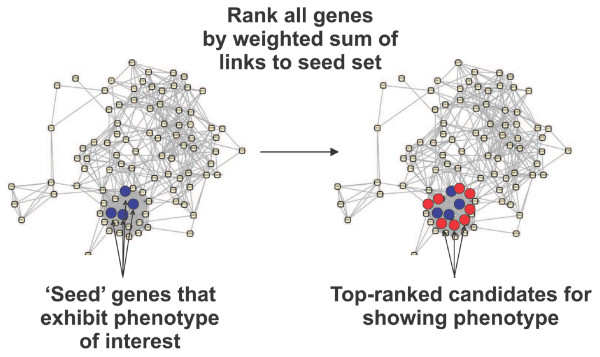
Overview of guilt-by-association phenotype prediction. Guilt-by-association phenotype prediction employs a functional gene network, represented here as circles (genes) connected by lines (functional linkages), and a seed set of genes (blue filled circles) whose disruption is known to give rise to the phenotype of interest. Neighboring genes in a functional gene network (red filled circles) are candidates for also giving rise to the phenotype. Candidates are prioritized by the sum of their network linkage weights to the set of seed genes. A gene strongly linked to multiple seed genes will thus rank more highly than a gene weakly linked to a single seed gene. Networks in Figures 1, 5, and 7 were drawn with Cytoscape [73].

In order to predict phenotypes, we took advantage of an established principle for inferring gene function from network connections, the principle of guilt-by-association (GBA). In GBA the function of uncharacterized genes is inferred from the functions of characterized neighbors in the network [9,21,23] (for review, see [19]). We employed GBA to consider whether the genes linked to a seed set of genes associated with a particular loss-of-function phenotype might also be more likely to result in the same phenotype upon disruption (Figure 1). For these analyses, we employ the most recent version (v. 2 [24]) of the probabilistic yeast functional gene network reported by Lee and coworkers [17]. This network describes 102,803 functional linkages among 5,483 yeast genes, each linkage scored with a probabilistic score capturing the tendency of the genes to share Gene Ontology (GO) 'biological process' annotation [24] versus prior expectation. Using this network, genes are rank ordered by the strengths of their linkages to the seed set; the genes linked most strongly to the seed set would therefore be considered candidates for leading to the same phenotype.

We first investigated whether the network could distinguish viable from nonviable yeast gene deletion strains. Essential genes of both yeast and humans are known to be more highly connected in protein physical interaction networks than nonessential genes [25-27], and there is evidence that essential proteins may also be enriched in the same physical complexes [28,29]. We considered whether essential genes could be predicted on the basis of their connections to other essential genes in a functional gene network. We employed the GBA approach, using as the seed set the 1,027 known essential yeast genes [4,30] and then scoring each gene in yeast for its likelihood to be essential as a function of connectivity to this seed set. Each gene in the seed set was withheld in turn from the seed set in order to evaluate it (performing leave-one-out cross-validation). As the prediction score for each gene, we calculated the sum of the weights of linkages connecting the query gene to genes in the seed set. Given that each linkage's weight in this network corresponds to the log likelihood of the linked genes belonging to the same pathway [24], the sum of linkage weights therefore represents the naïve Bayesian combination of evidence that the query gene belongs to the same pathway as the seed set genes. We expect genes in the same pathway often to exhibit the same loss-of-function phenotypes. Thus, this score should also serve to identify genes that share phenotypes with the seed set genes.

**Figure 2:**
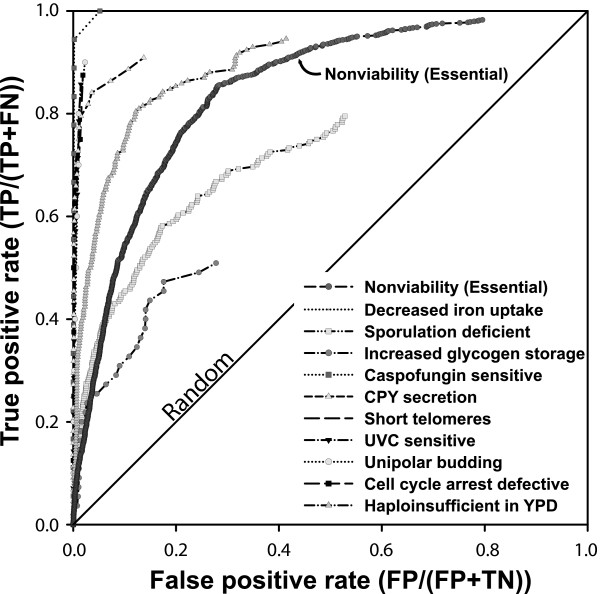
Diverse yeast gene loss-of-function phenotypes are predictable using guilt-by-association in a functional gene network. Predictability is measured in a receiver operating characteristic plot of the true positive rate (sensitivity) versus false positive rate (1 - specificity) for predicting genes giving rise to ten specific loss-of-function phenotypes, as well as for essential genes whose disruption produces nonviable yeast [4]. For each phenotype, each gene in the yeast genome was prioritized by the sum of the weights of its network linkages to the seed genes associated with the phenotype. Genes with higher scores are more tightly linked to the seed set and therefore more likely to give rise to the phenotype. Each phenotype was evaluated using leave-one-out cross-validation, omitting genes from the seed set for the purposes of evaluation. More predictable phenotypes tend toward the top-left corner of the graph; random predictability is indicated by the diagonal. For clarity, the line connecting the final point of each graph to the top right corner has been omitted. FN, false negative; FP, false positive; TN, true negative; TP, true positive.

To evaluate prediction quality, we calculated the true positive rate (sensitivity: TP/[TP + FN]) and the false positive rate (1 - specificity: FP/[FP + TN]), as a function of the prediction score, plotting the resulting receiver operating characteristic (ROC) curve. (The terms TP, FN, FP and TN mean true positives, false negatives, false positives and true negatives, respectively.) As Figure 2 shows, the essential genes are strongly predictable on the basis of their network neighbors. Therefore, in addition to the previous observations that essential genes have larger numbers of physical interaction partners, we demonstrate that essential yeast genes are also preferentially connected to each other in a functional network.

### A yeast gene network predicts varied, specific loss-of-function phenotypes

Although prediction of essential genes is useful (for example, for prioritizing knockout experiments or drug targets), there is far more utility in predicting highly specific phenotypes. *Saccharomyces cerevisiae *has been richly characterized, with a large number of systematically collected phenotypes, assayed across all (or, more typically, all nonessential) genes by taking advantage of yeast deletion strain collections [4,5]. In these collections, a single yeast gene is deleted in each yeast strain; a phenotypic assay on the complete set of knockout strains thereby associates that phenotype with those deleted genes that gave rise to it. These screens are ideal for addressing the general question of whether specific loss-of-function phenotypes are predictable. Importantly, the yeast gene network was neither trained on such data, and neither were phenotypic data incorporated into the network [24]. These sets are therefore fully independent test sets, and we could thus employ these data to evaluate the capacity of a gene network to predict loss-of-function phenotypes.

**Figure 3:**
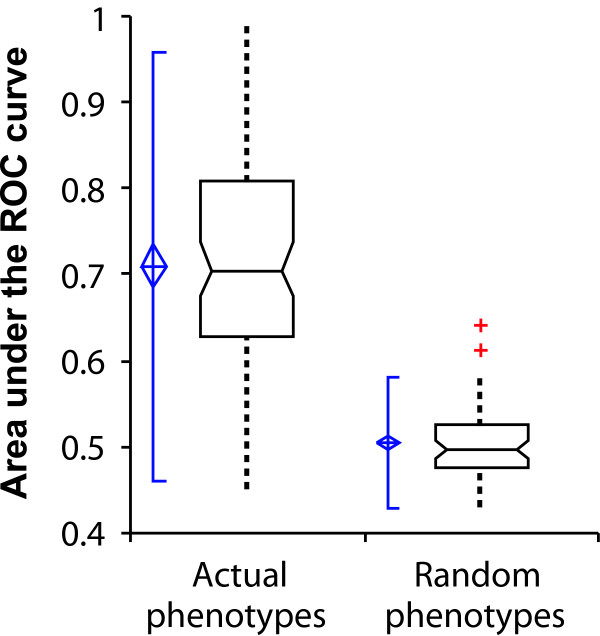
Loss-of-function phenotypes are predicted significantly better than random expectation. Here, predictability is measured as the area under a receiver operating characteristic (ROC) curve (AUC), measuring the AUC for each of 100 yeast phenotypes observed in genome-wide screens and plotting the resulting AUC distributions. Real phenotypes are significantly more predictable than size-matched random gene sets. At the left of each box-and-whisker plot, the center of the blue diamond indicates the AUC mean, the top and bottom of the diamond indicate the 95% confidence interval, and the accompanying solid vertical line indicates ± 2 standard deviations. The bottom, middle, and top horizontal lines of the box-and-whisker plots represent the first quartile, the median, and the third quartile of AUCs, respectively; whiskers indicate 1.5 times the interquartile range. Red plus signs represent individual outliers.

We assembled a set of 100 nonredundant phenotypes, either reported in the *Saccharomyces *Genome Database (SGD [31]) or in one of 32 additional publications in the literature (listed in full in Table 1). We evaluated each of the phenotypes for network-based predictability using ROC analysis, as shown for several examples in Figure 2. Specifically, we used hits from these screens as seed sets for predicting the associated phenotypes from the yeast network, performing leave-one-out cross-validation, just as for the prediction of essential genes. In order to evaluate the overall trends in these data, for each of the 100 ROC curves we calculated the area under the curve (AUC) as a measure of prediction strength; an AUC value of 0.5 indicates random performance, whereas an AUC value of 1.0 indicates perfect predictions. We find that a majority of phenotypes are reasonably predictable (Figure 3), with 70% of the phenotypes predictable at AUC above 0.65. In contrast, none of 100 random gene sets of the same sizes as the actual phenotypic seed sets exhibited AUC above 0.65. The AUC of the highest scoring random set was 0.64, which indicates that phenotypes with AUC above 0.65 were significant to at least *P *< 0.01.

**Table 1:** Predictability of 100 yeast gene deletion phenotypes

Phenotype^a^	AUC	Seed genes with phenotype (*n*)	Seed genes in network (*n*)	Ref.
Caspofungin sensitive	0.996	20	18	[36]
Increased resistance to calcofluor white	0.982	10	10	[33]
Unipolar budding	0.941	10	10	[68]
CPY secretion (3)	0.937	46	44	[34]
Cell cycle arrest defective	0.930	8	8	[74]
UVC sensitive (high)	0.919	15	14	[75]
Sensitivity at 15 generations in galactose	0.908	17	14	[4]
CANR mutator (high)	0.904	18	18	[76]
Haploinsufficient in rich medium (YPD)	0.898	184	184	[77]
Cellular chitin level increased (3)	0.873	22	21	[33]
Bleomycin resistant (3)	0.871	5	4	[37]
Morphology: branched (diploid)	0.870	5	5	[4]
Sensitivity at 15 generations in 1.5 M sorbitol	0.867	6	4	[4]
Caspofungin resistant	0.866	8	8	[36]
Inviable (essential)	0.845	1100	1027	[4,30]
Shortened telomeres (3)	0.843	20	18	[32]
Sensitivity at 15 generations in minimal +his +leu +ura medium	0.843	77	70	[4]
MMS sensitive (3)	0.837	78	73	[78]
Cellular chitin level reduced (2)	0.835	17	17	[33]
Petite	0.833	179	166	[79]
Sensitivity at 5 generations in minimal +his +leu +ura medium	0.827	62	51	[4]
Long telomeres (3)	0.824	6	6	[32]
Decreased calcofluor white resistance	0.814	65	63	[77,80]
Growth defect on a fermentable carbon source	0.812	257	249	[81]
Transposon cDNA expression changed (high)	0.810	27	26	[82]
Morphology: clumpy (3)(diploid)	0.802	18	18	[4]
Gamma radiation sensitive (3)	0.793	31	31	[83]
Cell cycle arrest defective and defective shmoo	0.782	30	29	[74]
Sensitivity at 5 generations in galactose	0.781	11	10	[4]
Small (haploid)	0.778	215	192	[84]
Retrotransposition reduced	0.772	99	89	[82]
K1 killer toxin sensitive (40%)	0.770	72	72	[80]
Increased iron uptake	0.757	76	70	[35]
Growth defect on a non-fermentable carbon source	0.755	498	448	[81]
Gentamycin sensitive (high)	0.754	11	11	[85]
Proteasome inhibitor sens (high)	0.753	22	22	[86]
Reduced fitness in rich medium (YPD)	0.748	891	872	[77]
Mycophenolic acid sensitive	0.746	38	33	[87]
Axial budding	0.745	4	4	[68]
Morphology: elongate (3) (diploid)	0.739	77	73	[4]
Sporulation deficient	0.738	261	244	[88]
Random budding (high)	0.737	74	72	[68]
Large (haploid)	0.728	227	205	[84]
Reduced sporulation (3) (normal respiration)	0.722	136	119	[89]
Bleomycin sensitive (4)	0.721	58	55	[37]
Sensitivity at 5 generations in synthetic complete - lys medium	0.715	23	22	[4]
Decreased rapamycin resistance	0.707	272	256	[90]
*Whi*	0.706	19	19	[79]
Sensitivity at 5 generations in 1.5 M sorbitol	0.704	13	11	[4]
Decreased wortmannin resistance	0.703	89	85	[90]
Sensitivity at 20 generations in 1 M NaCl	0.703	63	59	[4]
K1 killer toxin resistant (40%)	0.698	19	18	[80]
Morphology: round (3) (diploid)	0.696	105	99	[4]
*Uge*	0.694	28	26	[79]
Sensitivity at 5 generations in synthetic complete - trp medium	0.694	48	45	[4]
Sensitivity at 5 generations in 1 M NaCl	0.693	60	56	[4]
Rapamycin resist (2)	0.692	26	26	[91]
Reduced iron uptake	0.688	5	5	[35]
Rate of growth loss of growth in 0.85 M NaCl	0.682	212	189	[92]
Sensitivity at 5 generations in medium of pH 8	0.677	102	93	[4]
Sensitivity at 15 generations in medium of pH 8	0.676	128	115	[4]
Morphology: small (3)(diploid)	0.672	79	69	[4]
Sensitivity at 15 generations in 10 uM nystatin	0.672	28	27	[4]
Morphology: large (3)(diploid)	0.669	88	80	[4]
Reduced glycogen storage (2)	0.666	44	41	[93]
Sensitivity at 5 generations in 10 uM nystatin	0.666	124	108	[4]
Increased rapamycin resistance	0.662	114	100	[90]
Morphology: unusual shmoo (haploid)	0.661	29	25	[74]
Morphology: polarized bud growth (haploid)	0.657	5	5	[74]
Wortmannin resistant (5)	0.656	25	23	[94]
Sensitivity at 5 generations in synthetic complete - thr medium	0.647	31	29	[5]
Enhanced glycogen storage (2)	0.645	61	55	[93]
Proteasome inhibitor resistant	0.642	7	6	[86]
Reduced spores per ascus	0.641	37	34	[89]
Rate of growth sensitivity in 0.85 M NaCl	0.629	209	191	[92]
Morphology: football (3) (diploid)	0.628	59	53	[5]
Germination deficient	0.627	158	147	[88]
Sporulation promoting	0.622	102	98	[88]
6AU sensitive (3)	0.618	28	26	[95]
Increased wortmannin resistance	0.617	80	75	[90]
Morphology: elongated (haploid)	0.603	110	101	[74]
Rapamycin sensitive (4)	0.597	20	20	[91]
Efficiency of growth sensitivity in 0.85 M NaCl	0.597	65	58	[92]
Decreased rapamycin resistance	0.597	8	7	[90]
Slow growth in YPD (16× below WT)	0.585	23	22	[4]
MPA sensitive (3)	0.563	24	22	[95]
Morphology: round (haploid)	0.552	13	11	[74]
Efficiency of growth resistance in 0.85 M NaCl	0.541	44	40	[92]
Sensitivity at 5 generations in synthetic complete medium	0.531	88	78	[5]
Morphology: large (haploid)	0.527	23	21	[74]
Adaptation time loss of growth in 0.85 M NaCl	0.526	103	91	[92]
Adaptation time sensitivity in 0.85 M NaCl	0.521	284	259	[92]
Decreased sensitivity to the anticancer drug, cisplatin	0.512	22	19	[96]
Morphology: chain (diploid)	0.485	5	5	[5]
Morphology: small (haploid)	0.480	94	89	[74]
Rate of growth resistance in 0.85 M NaCl	0.479	59	49	[92]
Morphology: clumped (haploid)	0.479	32	28	[74]
Adaptation time resistance in 0.85 M NaCl	0.465	69	60	[92]
Efficiency of growth loss of growth in 0.85 M NaCl	0.464	23	21	[92]
Morphology: pointed (haploid)	0.453	99	88	[74]

^a^Numbers in parentheses indicate threshold applied to generate seed set; for instance, '(3)' indicates '+++' or '---', as appropriate.

**Figure 4:**
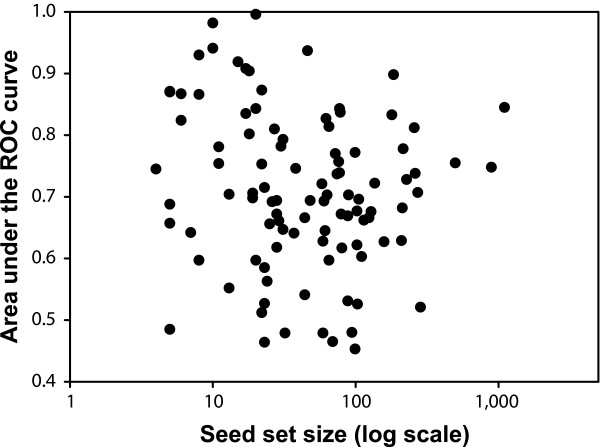
A plot of seed set size versus predictability of the phenotype shows no significant correlation. Thus, there does not appear to be an intrinsic limitation for applying network-guided reverse genetics even when seed set size is small. Each filled circle indicates the prediction strength (area under the receiver operating characteristic [ROC] curve, as calculated in Figure 3) of one of the 100 loss-of-function phenotypes relative to the number of genes in that seed set.

The most strongly predictable phenotypes vary widely in specificity and character. For example, we observed strong predictability for genes whose disruption leads to shortened telomeres [32], causes chitin accumulation [33], or increases secretion of the vacuolar protein carboxypeptidase Y [34]. Even gross cellular morphologies (small cells, round cells, and so on) are somewhat predictable, as are far more specific phenotypes, such as increased iron uptake [35] and caspofungin sensitivity [36]. Surprisingly, there is little dependence of predictability on the size of the seed set (Figure 4), and we observed strong predictability for both large and small seed sets (for example, bleomycin resistance [37] [four genes, AUC = 0.87] versus nonviability/essential [4,30] [1,027 genes, AUC = 0.85]).

### Integration of functional genomics and proteomics data is important for phenotype prediction

Because physically interacting proteins often share related genetic interaction partners (for examples, see [38,39]) and even human disease associations [25,40,41], it seemed likely that physical protein interactions might account for a large fraction of the signal we observe. In particular, Lage and coworkers [40] used GBA among protein complexes to predict disease genes within human genetic linkage groups. Balancing this trend, phenotypes of annotated genes are in part predictable directly from their functional annotations [42]. Thus, we considered whether the integration of functional genomics and proteomics data in the functional network yielded additional predictive power over physical interactions alone. We measured the median AUC across the 100 phenotypes for the functional yeast gene network and for each of several published versions of the yeast protein physical interaction network [29,43-45]. We compared these values with the median fraction of each seed gene set covered by the respective networks. The values of AUC and fraction covered therefore serve as measures of precision and recall for each network.

**Figure 5:**
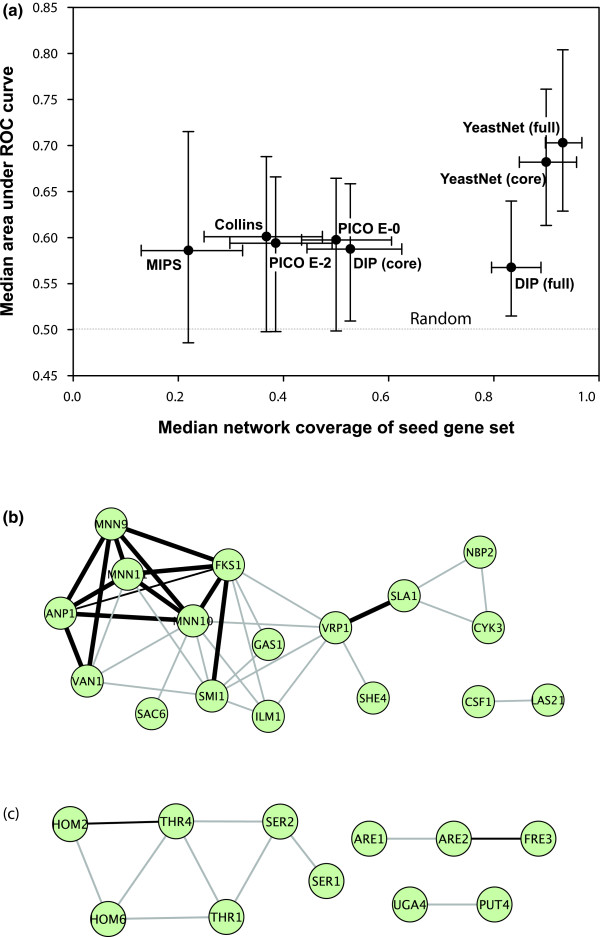
Relative predictive power of functional and physical protein networks. (a) Median values of predictive power (area under the receiver operating characteristic [ROC] curve [AUC]) across 100 loss-of-function phenotypes are plotted versus the median fraction of each seed gene set covered by a network (coverage; measured as the fraction of seed genes with at least one linkage in the network). Five networks are compared: the functional yeast network (YeastNet v. 2 [24]) and four versions of the network of yeast physical protein interactions (Database of Interacting Proteins [DIP] [45], Probabilistic Integrated Co-complex [PICO] [29], Munich Information Center for Protein Sequences [MIPS] physical complexes [44], and Collins and coworkers [43]). DIP, PICO, and YeastNet are each evaluated at two reported confidence thresholds. The YeastNet functional gene network shows considerably higher predictive power than for the networks composed only of physical interactions; the full YeastNet shows higher predictive power than a more confident core set of the top 47,000 linkages, indicating that the lower confidence linkages nonetheless add predictive power. Error bars indicate the first and third quartiles. Panels b and c show example seed gene sets (green circles) and their network connections, indicating functional linkages in grey lines, physical interactions in thin black lines, and both functional and physical interactions in thick black lines. **(b) **Genes whose deletion increases cellular chitin levels [33] (AUC = 0.87), whose prediction relies upon a mix of physical and functional interactions. **(c) **Genes whose deletion confers sensitivity at 5 generations in synthetic complete medium lacking threonine [4] (AUC = 0.65), whose prediction derives predominantly from functional linkages.

**Figure 6:**
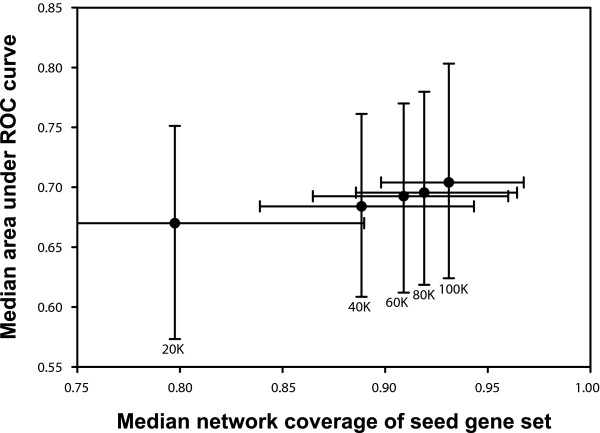
Lower probability linkages continue to improve predictive accuracy. The continued improvement of predictions, albeit with diminishing returns, is shown in a plot of the predictive accuracy (median area under the receiver operating characteristic [ROC] curve across the 100 phenotypes, calculated as in Figure 3) versus median network coverage of the 100 phenotype sets, as calculated for the top-ranked 20,000 (20 K), 40,000 (40 K), 60,000 (60 K), 80,000 (80 K), and 100,000 (100 K) linkages in YeastNet v. 2. This trend derives from the fact that all links in this network have at least a 60% probability of linking genes in the same pathway. The probabilistic nature of the network means that low confidence linkages are unlikely to undercut high confidence linkages during phenotype prediction because the links are weighted according to the strength of the evidence supporting them. Error bars indicate the first and third quartiles.

As Figure 5 demonstrates, we observe that all networks predict loss-of-function phenotypes to some extent, but find the functional network to predict phenotypes at a significantly higher precision and recall. We attribute this enhanced performance to the increased comprehensiveness of the functional gene network, both in terms of additional types of gene associations and more extensive coverage of the overall set of yeast genes. The functional network accomplishes this by incorporating other sources of functional interaction (for example, mRNA co-expression) in addition to physical interactions from both small-scale (for example, the Database of Interacting Proteins [DIP] and Munich Information Center for Protein Sequences [MIPS] databases) and genome scale (for example, mass spectrometry of affinity-purified protein complexes and yeast two hybrid) experiments. Furthermore, as shown in Figure 6, the sequential addition of progressively lower confidence functional linkages increases both predictive accuracy and coverage. Low confidence linkages do not undercut the predictive power of high confidence linkages because they are weighted in proportion to the strength of the evidence that supports them. These observations highlight the importance of integrating diverse data types into gene networks for the purposes of predicting phenotypes and suggest that the proteins encoded by genes associated with the same phenotype often may not physically interact.

### Extending a genetic screen by network-guided reverse genetics

**Figure 7:**
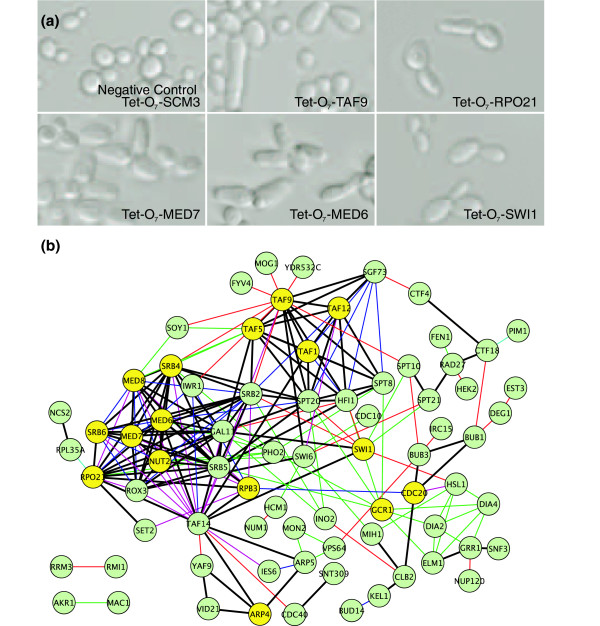
Network-guided extension of a genetic screen. Guilt-by-association (GBA) was applied to predict essential yeast genes whose disruption resulted in elongated yeast cells, based on the genes' network connectivity to a seed set of 77 nonessential genes already known to cause cell elongation when deleted [4]. **(a) **Five examples of successful predictions, observed in yeast strains carrying tetracycline downregulatable conditional alleles [47] of the essential genes *TAF9*, *MED6*, *MED7*, *SWI1*, and *RPO21*. In contrast, conditional downregulation of an unrelated essential gene, *SCM3*, caused no such cell elongation. **(b) **Sixteen out of 33 tested essential genes (yellow circles) showed elongated cell phenotypes on the basis of their connections to the seed set genes (green circles), with particular enrichment for genes associated with RNA polymerase II transcriptional initiation and the mediator complex. The color of the edge between two genes indicates the source of evidence supporting the functional link: thick black, multiple types of evidence; blue, affinity purification/mass spectrometry; green, literature mining by co-citation; cyan, gene neighbors or tertiary structure; pink, literature curated physical interaction; and red, genetic interaction.

For organisms in which reverse genetics is feasible, the observation that phenotypes can be predicted from network connectivity opens the possibility of extending genetic screens in a directed manner. That is, when in possession of a set of genes known to give rise to a phenotype of interest, rather than randomly screening to identify additional genes, one could instead exploit the predictability of phenotypes by directly screening genes that are most strongly connected to the known set in the network. In this manner, experiments could be focused on the genes that are most likely to give rise to the phenotype. We tested this notion for yeast genes whose disruption gives rise to a simple cell morphology defect, the formation of elongated yeast cells. Across the complete set of nonessential genes, 145 genes (3.3%) have been identified that give rise to elongated morphologies in homozygous diploid deletion strains, of which 77 genes (1.7%) show a strong phenotype [4]. We selected these 77 genes as a seed set and found the phenotype to be reasonably predictable from the network using ROC analysis (AUC = 0.74). Because the complete set of nonessential genes was previously screened for cell morphology defects [4,46], we instead considered which essential genes were most strongly linked to the seed set, selecting the top-ranked 35 essential genes for further evaluation, and tested 33 of these strains. We examined conditional loss-of-function strains for elongated cell morphologies, performing light microscopy of yeast strains carrying tetracycline downregulatable alleles for each candidate gene [47]. Sixteen (about 48%) of the 33 tested were elongated, as shown for several examples in Figure 7. As negative controls, we tested 17 strains carrying tetracycline downregulatable essential genes that were chosen for being unlinked in the functional network to the seed set. One negative control strain also scored as elongated; this strain had also been previously identified as such by Mnaimneh and coworkers [47]. The results represent an eightfold improvement over the negative control set and a more than 15-fold improvement over genome-wide screening, validating the general strategy of network-guided genetic screening.

To gain further insight into the genes identified, we examined the network connections among the seed genes and newly identified genes giving rise to the elongated phenotype (Figure 7b). Strikingly, the genes associated with elongated yeast cell morphology are strongly enriched for core transcriptional functions (for example, they are significantly enriched for the MIPS [48] annotation 'mRNA synthesis';*P *< 10^-12 ^[49]), with the set of newly identified genes predominantly belonging to the RNA polymerase II mediator complex and associated transcriptional machinery. In particular, the directed screen identified the genes *MED6*, *MED7 *(confirming an earlier observation reported by Boone and coworkers [47]), and *MED8*, all of which are core components of the mediator complex. It also identified the genes *TAF1*, *TAF5*, *TAF9*, and *TAF12*, all of which are subunits of the TFIID and SAGA transcriptional complexes, which are required for RNA polymerase II transcriptional initiation. These findings highlight another advantage of network-guided genetic screening; because candidate genes are selected directly from the gene network, functional connections are often already known among the genes, helping to guide later interpretation of the hits. The findings also highlight the often mysterious relationship between an observed phenotype and the corresponding molecular defect. The mechanism is unknown by which defects in transcription initiation lead to elongated cells; nonetheless, the relationship is robust enough that genes whose disruption causes cell elongation can be correctly predicted.

### Prediction of quantitative cell morphology phenotypes

Given that the phenotypes analyzed thus far are often based on subjective criteria (judged to be elongated or not), it is important to consider whether such predictions can be made for quantitative phenotypes. We therefore examined quantitative cell shape data that were recently systematically measured for the set of haploid MATa yeast deletion strains [46]. A total of 281 quantitative features of cell shape, cellular, and subcellular morphology were measured for each strain, including such parameters as the ratio of long cell axis to short cell axis, the angle between a mother cell and bud, and the relative distribution of actin with regards to the bud position. Each feature was measured for many cells from a given strain, and the mean value reported. For 220 of the features, the coefficient of variance (CV) was also reported, describing the variability in that feature across single cells in that strain. Considering the mean value of each feature and the CV as separate traits (we refer to the former as morphology phenotypes and the latter as CV phenotypes) means that a total of 501 cell shape measurements or CVs were reported for 4,718 strains, and made available through the *S. cerevisiae *Morphology Database (SCMD) [50]. Because not all measurable cell shape features are likely to be under selection (for example, they might simply vary stochastically yet neutrally), we do not expect all such phenotypes to correspond to functional pathways and therefore be predictable. Nonetheless, we might expect that a number of these would have functional correlates and therefore be predictable. In order to test this notion, we therefore evaluated each of the 501 features for predictability using the functional gene network.

**Figure 8:**
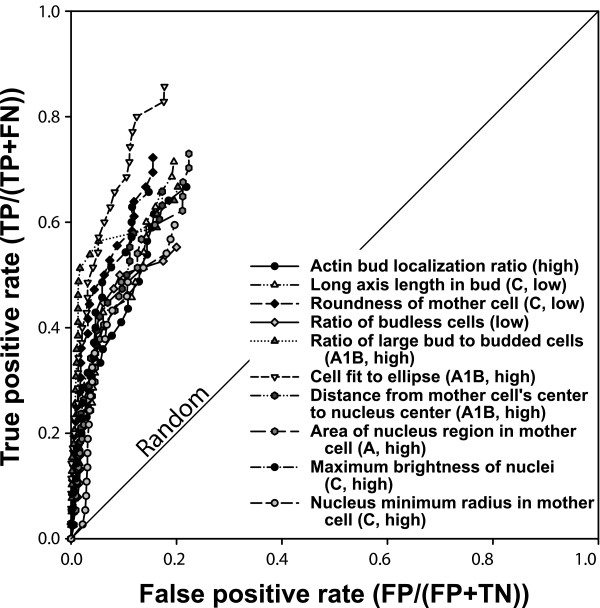
Network-based prediction of quantitative cell morphology phenotypes. A wide variety of phenotypes based upon quantitative yeast cell shape and intracellular features [46] are predictable, as shown for the ten phenotypes in this receiver operating characteristic (ROC) analysis (selected from *S. cerevisiae *Morphology Database [SCMD] phenotypes with area under the ROC curve [AUC] > 0.68). For each of the features, the 40 genes whose deletion mutants show either the 40 highest or 40 lowest values for that quantitative feature (indicated by 'high' or 'low', respectively) were selected as the seed gene set. Predictability was evaluated using ROC analysis as in Figure 2, plotting the true positive prediction rate versus false positive rate, using leave-one-out cross-validation. For clarity, the line connecting the final point of each graph to the top right corner has been omitted. Labels of features are adapted for clarity from the SCMD [50]; the SCMD labels A, A1B, and C represent unbudded cells, budded cell with one nucleus in mother cell, and large-budded post-mitotic cells with nuclei in both mother and daughter cell, respectively. Ratio measurements refer to proportions across a population of cells. FN, false negative; FP, false positive; TN, true negative; TP, true positive.

**Figure 9:**
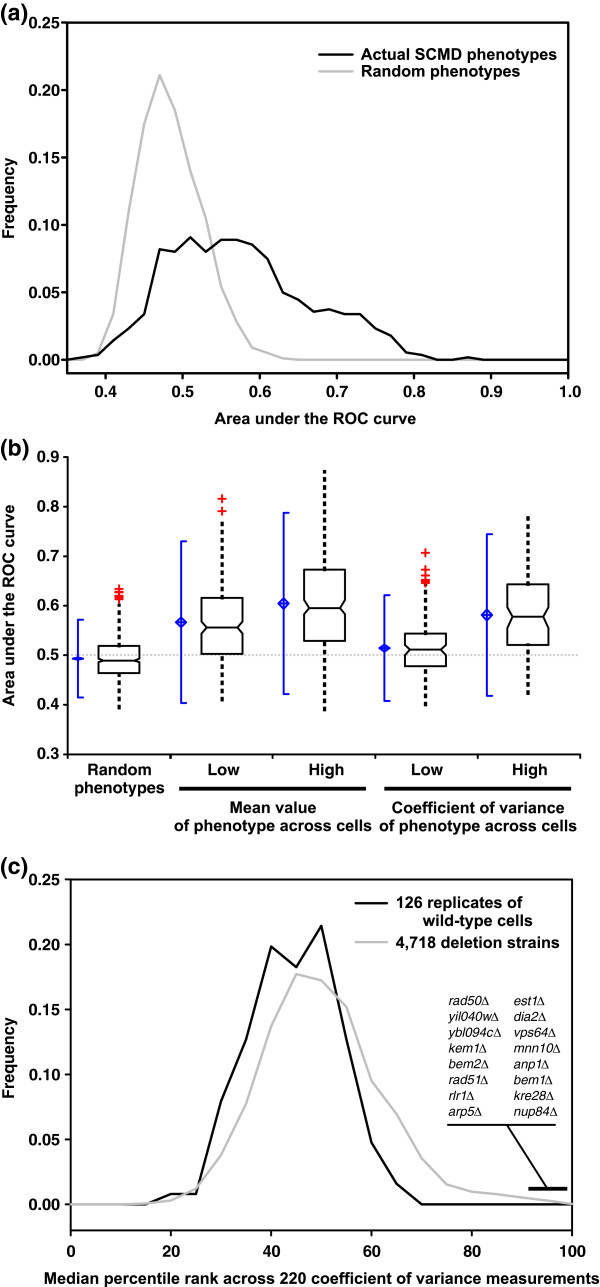
Quantitative cell morphology phenotypes are predicted significantly better than random expectation. In contrast, genes whose disruption decreases population co-efficient of variance (CV) were not predictable. **(a) **A histogram plotting the distribution of the area under the receiver operating characteristic (ROC) curve (AUC) values for 562 quantitative morphological phenotypes shows a significantly higher proportion of high AUC values than for 1,000 size-matched random gene sets. **(b) **Separate analyses of phenotypes associated with morphologic features and phenotypes associated with cell-to-cell variability in the morphologic features reveals asymmetry in predictability. Sets of genes whose disruption causes the 40 largest or smallest mean values of a morphological feature (middle plots) are significantly more predictable than random gene sets (left side). By contrast, although the sets of genes whose disruption most increase the CV tend to be predictable (high AUC), those that most decrease the CV are not (low AUC). Box-and-whisker plots are drawn as in Figure 3. **(c) **A comparison of the median phenotypic CVs observed for deletion strains versus replicate analyses of wild-type cells shows that deletion strains with the most reduced CVs are essentially wild-type-like in character, whereas those with the most increased CVs show significantly more cell-to-cell variability than wild-type cells. These latter knockout strains carry deletions for genes predominantly involved in maintaining genomic integrity. This trend is therefore likely to have arisen from nonclonal genetic variation in these strains, recapitulating the classic mutator phenotype.

To generate seed gene sets from these data, for each of the 281 quantitative features we selected as phenotypic seed sets the 40 genes with the highest measured mean value of that feature and the 40 genes with the lowest measured mean value of that feature, in all generating 562 morphology phenotype seed gene sets (281 features × 2 seed sets each). We then evaluated each of these seed sets for predictability using ROC analysis. As for the 100 genome-wide phenotypic screens, we observed many strongly predictable cell morphology phenotypes, such as those illustrated in Figure 8. For example, one of the most strongly predictable cell morphology phenotypes is for the genes whose disruption most increases cell ellipticity during nuclear migration to the bud neck (AUC = 0.87). Another strongly predictable phenotype is for deletion strains showing the highest increase in the actin polarization of unbudded cells (AUC = 0.80). We observe the overall set of cell morphology phenotypes to be significantly more predictable than random expectation, as shown by comparison of the distribution of AUC values with those derived from 1,000 random seed sets of 40 genes each (Figure 9a). Note that predictability does not depend strongly on the size of the seed sets; we see similar predictive power with seed sets of 10 to 80 genes (data not shown). These findings confirm that even specific quantitative aspects of yeast cell shape often have functional correlates, and therefore the sets of genes whose disruption most affects such features are predictable.

### Genes increasing cell-to-cell variation are less functionally coherent than those decreasing variation

Because the SCMD data include both morphology features and measurements of their cell-to-cell variability, we considered more specifically whether the CV of a yeast morphology phenotype across single cells in a population was itself a predictable phenotype. Strikingly, we observed good predictability for sets of genes whose disruption most increased the CV of a given morphologic feature (for instance, the 40 genes whose deletion caused the highest increase in bud neck width CV; AUC = 0.70), but near random prediction for sets of genes whose disruption most decreased the CV in a given morphologic feature (for example, the 40 genes whose deletion most reduced bud neck width CV; AUC = 0.54; Figure 9b). The high CV phenotypes are significantly more predictable than the low CV phenotypes (*P *< 0.0001, Wilcoxon signed-ranks test). Across the 220 high CV phenotypes, we observed 116 to exhibit significantly greater AUC values than size-matched random sets (at the 95% confidence level, as judged by Z-score > 1.95), whereas only 26 of the set of 220 low CV phenotypes were better than random at this level.

Because successful prediction of a loss-of-function phenotype implies functional coherence of the genes - essentially reflecting clustering of the genes in the functional network - this result indicates that the genes whose disruption most strongly reduced the CV in a given morphologic feature do not in general form a functionally coherent set. By contrast, genes whose disruption most increased morphologic phenotypic variability were predictable, and thus functionally coherent. We further observed that the same genes tended to be present in the phenotypic sets from many different CV phenotypes; namely, there are particular genes whose deletion increases the CV of a large number of otherwise unrelated morphologic properties.

To explore this observation further, for each of the 4,718 yeast genes in the SCMD data set, we calculated the median percentile rank across each of the 220 SCMD CV phenotypes. Thus, the gene whose deletion strain has the highest median percentile rank (the telomere length regulation gene *EST1*; median percentile rank of 0.98) exhibits the greatest cell-to-cell variability across nearly all of the set of 220 CV phenotypes. By contrast, the gene whose deletion strain has the lowest median percentile rank (*YAL004W*, a small open reading frame that overlaps the coding sequence for the heat shock protein 70 family chaperone SSA1; median percentile rank 0.17) consistently exhibits the lowest cell-to-cell variability for the tested phenotypes. Thus, these rankings capture the generic tendency for a gene to increase or decrease cell-to-cell variability across many measured morphology parameters. We tested the top-ranked 40 genes and the bottom-ranked 40 genes for their network-based predictability.

As with our earlier observations, the top-ranked 40 genes (those with highest median percentile rank) exhibit reasonable predictability (AUC = 0.71), whereas the bottom-ranked 40 genes exhibit random predictability (AUC = 0.49). Thus, either on a phenotype-by-phenotype basis, or across all 220 phenotypes, genes whose disruption most increased morphologic phenotypic variability tended to be more predictable and functionally coherent than those that reduced phenotypic variability. An examination of the functions of the top-ranked 40 genes suggested a possible explanation. The top-ranked set show strong enrichment for specific GO terms, with 17 of the 40 genes encoding nuclear proteins (*P *< 10^-6^; measured using FunSpec [49]); ten of these are DNA-binding proteins (*P *< 10^-4^), including genes of DNA recombination and repair (*P *< 10^-6^). Among these genes are many that are involved in maintaining genomic stability, including the repair/recombination proteins RAD27, RAD50, RAD51, RAD52, CTF4, HEX3, RTT109, and THP1, the histone HTZ1, and the telomere maintenance protein EST1. Thus, although deletions of these genes may possibly increase phenotypic variation, a more likely possibility is that these particular strains in the yeast deletion collection have accumulated genetic variation and are no longer clonal, as we discuss below.

### The functional network predicts yeast orthologs of human disease genes

**Figure 10:**
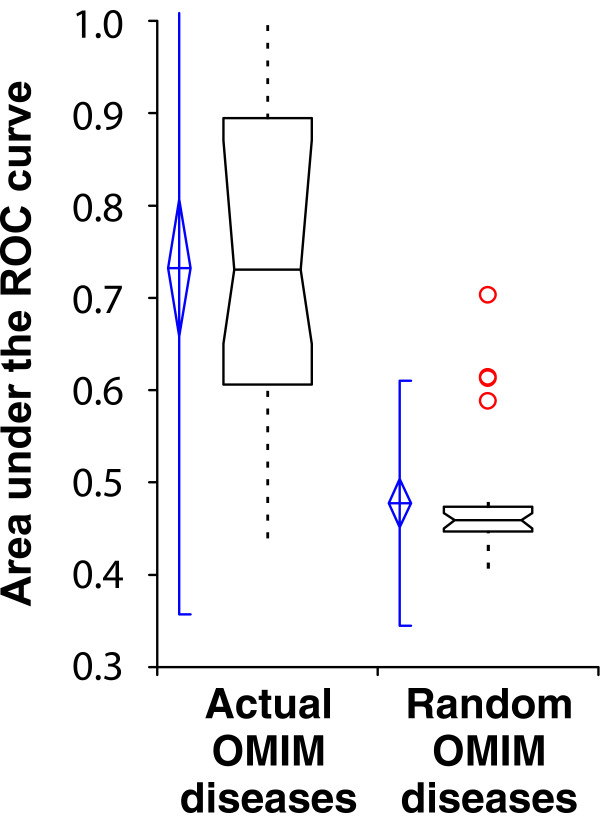
Yeast genes with human orthologs linked to the same diseases are predicted better than random expectation. Predictability is measured as the area under a receiver operating characteristic (ROC) curve (AUC), as in Figure 3, measuring the AUC for each of 28 human diseases reported in the Online Mendelian Inheritance in Man (OMIM) disease database [51] that have four or more yeast orthologs annotated in the yeast function network and plotting the resulting AUC distributions. Real disease gene sets are significantly more predictable than size-matched random gene sets drawn from the set of yeast-human orthologs. Box plots are drawn as in Figure 3.

The network's effectiveness at predicting both qualitative and quantitative yeast phenotypes suggests the possibility of application to other organisms, such as for predicting human disease genes. We tested the potential of this approach by examining the power of the yeast network to predict yeast orthologs of human disease genes, focusing on all human diseases listed in the Online Mendelian Inheritance in Man (OMIM) disease database [51], for which at least four yeast orthologs existed in YeastNet. We observed strong predictability for the majority of the 28 human diseases that could be tested in this manner, as shown in Figure 10. Not only are many of the yeast orthologs of these disease genes predictable, but also the median predictive accuracy of these phenotypes is even slightly higher than the genome-wide yeast phenotypes (Figure 3). This is a probable reflection of the fact that genes conserved between yeast and humans generally compose core cellular machinery, well captured by the gene network. For example, the most predictable disease we observed (AUC = 1.0) was leukoencephalopathy with vanishing white matter, arising as the result of mutations in any of the subunits of the translation initiation factor EIF2B. Likewise, we observed strong predictability for hemolytic anemia (AUC = 0.89), which involves 11 ortholog groups, involved in glycolysis and glutathione metabolism, which are linked primarily by co-expression and co-citation, with only a few physical interaction-based linkages.

Although this test was limited to diseases involving biologic processes shared between human and yeast, these results support the notion that an integrated human functional network would guide the discovery of new disease genes. Because we observe strong disease predictions both from protein complexes (as in leukoencephalopathy) and pathways (as in hemolytic anemia), it appears likely that a functional human gene network might offer strong predictions for genes associated with diverse human diseases, even in the absence of genetic linkage data.

## Discussion

Just as functional networks propagate known functional annotations to un-annotated genes, phenotype prediction via GBA is limited to propagating known phenotypes. Therefore, an initial seed set of genes is required, such as might result from a genetic screen for the phenotype of interest, before being able to apply the network in order to identify more such genes. We might also expect genes in the same pathway often to exert inverse effects on a phenotype, acting either as activators or repressors. Nonetheless, we demonstrate that GBA can successfully be applied to identify genes that give rise to similar loss-of-function phenotypes. Furthermore, network-guided phenotype prediction can be used to extend a genetic screen in a targeted manner by providing a ranked list of potential candidates for evaluation. In principle, the screen might be expanded by adding the newly identified genes to the seed set and iterating the prediction and testing.

In particular, large-scale reverse genetic screens using yeast mutant strain collections have become increasingly common [52]. However, these assays often suffer from high false negative rates, not least by virtue of screening libraries of limited scope (for example, screening only the nonessential or essential genes). Such partially genome-wide screens can benefit by following up the initial screen with focused screening (or re-screening) of prioritized candidate genes. In order to facilitate such efforts, we have created a web server [53] that allows interactive analysis of a seed gene set, performing ROC analysis to assess the predictability of the phenotype, then returning a ranked list of candidate genes that are most likely to share the same loss-of-function phenotype.

Note that we have focused here on predicting loss-of-function phenotypes because of the large number of genome-wide screens available; it is not clear that gain-of-function phenotypes will be similarly predictable. However, the recent construction of yeast over-expression libraries [54-56] should soon allow testing of network-based prediction of such phenotypes.

### Why are loss-of-function phenotypes predictable?

Our findings indicate that typical phenotypes represent specific enough defects that they are predictable based upon the genes' functional associations. We observe multiple mechanisms for how loss of different genes leads to disruption of the same phenotypically relevant process, primarily participation in the same protein complex or membership in the same biologic pathway. These results are consistent with the partial predictability of human disease from protein complex membership [40,41] and of the prediction of knockout phenotypes of annotated yeast genes on the basis of pathway annotation [42], which we illustrate with the following contrasting examples from among our predictions. In Figure 5b, the proteins ANP1, MNN9, MNN10, MNN11, VAN1 are members of the same α-1,6-mannosyltransferase protein complex. Chitin accumulates when the function of the complex is disrupted by the loss of any one of the five members [33]. In contrast, in Figure 5c the three genes *THR1*, *THR4*, and *HOM6 *are involved in the biochemical pathway that converts homoserine to threonine. These genes are linked in the functional network [24] by virtue of the coordinate expression of their bacterial homologs in operons (for example, as for the *Bacillus subtilis *homologs ThrB, ThrC, and ThrA), even though there is as yet little evidence that they belong to the same physical complex. The loss of any of the three genes disrupts the threonine synthesis pathway and leads to reduced growth after five generations in threonine-depleted media [4]. The functional gene network, which combines both physical and functional interactions, predicts both classes of phenotypes effectively, whether resulting from disruption of physical complexes or pathways.

Nevertheless, some phenotypes are not significantly predictable. Three likely causes exist. First, poor predictability may result from using genome-wide screens with high false positive rates, which would base predictions on incorrectly identified seed sets. We sought to minimize this type of error by adopting stringent thresholds for each phenotype. Second, incomplete screens (such as by not testing the essential genes), high false negative rates, and the stringent phenotype thresholds that we selected could lead to a large number of positive examples being excluded from the seed sets. Such omitted positive examples scoring higher than seed genes would artificially depress prediction accuracies. Third, unpredictable phenotypes could in principle arise from the disruption of functionally unrelated genes. In order to test this, we compared the GO enrichment for the 25 most predictable phenotypes with the 25 least predictable phenotypes. For each phenotype, we identified the GO term with the most significant enrichment of genes annotated with the term, measured using the hypergeometric distribution. Using a significance threshold of *P *< 10^-7^, we find that 18 of the 25 highly predictable phenotypes are significantly enriched for at least one GO annotation, as compared with only two of the 25 poorly predictable phenotypes. This suggests that poorly predictable phenotypes largely result from sets of genes with little functional coherence.

### AUC is a useful measure of gene functional coherence

By definition, the GBA approach we present predicts phenotypes associated with functionally coherent sets of genes, presumably reflecting the clustering of the genes in the functional network. Such predictability, which we specifically measure as the AUC, can therefore be regarded as a direct estimate of the functional coherence of the seed gene set. Thus, beyond simply evaluating phenotype prediction, the AUC offers an additional measure of functional coherence that complements other existing measures, such as the enrichment of GO annotations or other biologically meaningful sets of genes (as calculated by FunSpec [49] and Database for Annotation, Visualization, and Integrated Discovery [57]). For example, the five genes giving rise to the branched cell phenotype are connected by six linkages in the network (AUC = 0.87), but only a single pair shares any GO annotation (*P *< 0.001, for the GO term 'transcription from RNA polymerase II promoter'). The network-based AUC measure for functional coherence exploits the massive unbiased data integration of functional networks, extending well beyond known annotations, and allows estimates of functional coherence even among unannotated genes or those spanning multiple systems.

In principle, the AUC approach can therefore measure the functional coherence of genes that annotation-based methods will miss. Beyond un-annotated genes, the AUC-based estimate of functional coherence might also work effectively when the genes under study span multiple functional categories; each category may be only partially enriched and therefore may otherwise be missed for lack of signal. The functional network, however, considers pair-wise linkages, not predetermined categories, and so it has the potential to identify linked genes across multiple annotation categories.

### Recapitulation of the classic mutator phenotype in the yeast knockout collection

We observed a strikingly higher predictability for mutations that increased cell-to-cell phenotypic variation versus those that decreased it. The deletion strains exhibiting higher CVs tended to be consistent across the complete set of CV phenotypes examined, with the deleted genes showing strong enrichment for functions related to DNA repair, recombination, and genomic stability. Note that strains with the lowest CV phenotypes exhibited neither predictability nor functional enrichment; in fact, the CVs exhibited by these strains were similar to those observed for replicate analyses of wild-type cells (Figure 9c). This suggests that the strains that most decreased cell-to-cell variation were essentially wild-type-like in this regard.

This outcome is consistent with a recapitulation in the yeast deletion strain collection of the classic mutator phenotype. The mutator phenotype was originally observed in DNA repair mutants; such mutants accumulated mutations so rapidly that they showed high variability in colony sizes when grown on Petri dishes, high variability in cell morphologies, high rates of plasmid loss, and increased spontaneous mutagenesis (for example, as previously observed for RAD27 and RAD52 deletion mutants [58,59]). The most likely explanation is therefore that strains in the deletion collection harboring deletions in genes related to genomic stability have simply accumulated mutations at a higher rate. A mixed population, no longer clonal, would be expected to exhibit more cell-to-cell variation than other deletion strains, which would accumulate mutations at a lower rate. Thus, we suspect that our phenotypic analysis is correctly revealing the functional signature of a legitimate phenotype inadvertently captured in the process of distributing and passaging the yeast deletion strain collection.

### Applying network-based phenotype prediction to humans and other organisms

In principal, the approach we describe could be applied to any organism, using functional network data if available or, in the absence of such data, using physical interaction data, such as available protein interaction networks for fly [60], worm [61], or human [25,62-66]. In the absence of an integrated functional gene network or protein interaction network, we expect that networks of mRNA co-expression associations, such as can be derived from DNA microarray data, would provide some utility for phenotype prediction. Such data are a major contributor to functional gene networks (for examples, see [13,16,17]) and are relatively easily generated from available data for most model organisms.

In particular, application of this approach in humans may allow directed identification of disease genes. Indeed, functional linkages derived largely from known GO annotation [67] or protein interactions [40] have shown some utility for prioritizing positional candidate genes from genome-wide linkage screens. However, our results show that across a wide range of yeast phenotypes and human diseases the associated genes (or their yeast orthologs) can be directly identified even in the absence of supporting genetic loci data. In order to apply our approach to human diseases, genes that are known to be associated with a particular disease, such as found from twin or genome-wide association studies, would form the seed set. Additional candidate genes that are likely to be associated with that disease could then potentially be identified or prioritized based upon their network connections to the seed set, using the GBA principle. Potential disease genes could then be tested in disease model systems or screened genetically in a focused manner. Such a directed approach would exploit the tremendous existing body of knowledge about protein interactions and functional pathways.

## Conclusion

We have demonstrated that yeast gene loss-of-function phenotypes are broadly predictable from connectivity in a functional gene network, with examples presented spanning a wide range of cell growth, cell morphology, metabolite transport, chemical sensitivity, and molecular phenotypes. We demonstrate that this predictability can be used to extend genetic screens in a directed fashion, and that this approach might therefore be important in organisms for which genetics is difficult. We suggest that a similar approach in humans might enable the directed discovery of disease genes.

## Materials and methods

### Assembling the set of nonredundant loss-of-function phenotypes

A literature search was conducted to find genome-scale studies of yeast gene knockout phenotypes. Datasets were compiled from studies that systematically examined a large fraction of the yeast genome. No effort was made to minimize redundancy among the gene sets themselves. Nonetheless, only one set is a strict subset of another (genes that have changed levels of transposon cDNA upon knockout are a subset of the genes that reduce retrotransposition). Most studies were conducted using one or more of the following strain collections: haploid, homozygous diploid or heterozygous diploid [4], or tetracycline titratable [47]. The reported data were a mix of qualitative, pseudo-quantitative, and quantitative results. Pseudo-quantitative data (often reported as '+', '++', '-', '--', and so on) were thresholded at the most stringent reported value (except for the small set of genes conferring the phenotype 'branched cells' [4]; all genes with this morphology were included). Quantitative data were arbitrarily thresholded using cut-offs that appeared consistent with the sensitivity of the assay. Predictability was not used as a criterion for selecting thresholds. In some cases, thresholds less stringent than those selected result in more predictable phenotype sets (data not shown). In cases in which an uncharacterized open reading frame overlapped a known gene on the chromosome and both shared the same phenotoype (for instance, Axial budding [68]; the dubious open reading frame YOR300W overlaps BUD7), the uncharacterized gene was removed from the phenotype set. Additional phenotypes were collected from the SGD stabase [69]; phenotypes extracted from SGD used the threshold determined by SGD. The complete set of 100 phenotypic seed sets is provided as Additional data file 1.

For the 281 quantitative phenotypes reported by SCMD [50], the 40 knockout strains with either the highest or lowest values for each SCMD feature were selected (resulting in 562 seed gene sets). Similarly, 440 CV phenotypes were generated by considering the 40 knockout strains with either the higher or lowest CV for each SCMD CV feature (220 total features).

### Prediction of phenotypes and evaluation of prediction quality

For each gene in the network, we calculated the sum of its link weights to genes with the phenotype in question (the seed set), namely assigning each gene *i *the following score:

$$S_{i}=\sum_{j\in seed}LLS_{ij}$$

Where *j *is a gene in the seed gene set and *LLS*_*ij *_is the log likelihood score for the linkage between genes *i *and *j*, as reported by Lee and coworkers [24], except where explicitly analyzing other networks. Genes were then rank-ordered by their *S*_*i *_scores, with the highest scoring genes being the ones most likely to share the phenotype with the seed set. For networks reporting only binary linkages (MIPS [44] and DIP [45]), we considered all linkages to be of weight 1. For calculation of Figure 5, YeastNet v. 2, DIP and Probabilistic Integrated Co-complex (PICO) [29] were each evaluated at two different confidence levels. For analyses of protein interaction networks, the following networks were analyzed: YeastNet v. 2, which corresponds to all interactions reported by Lee and coworkers [24]; physical protein interactions (PPIs) from the DIP [45] (downloaded on 4 February 2007), selecting as the core set those interactions reported by Deane and coworkers [70]; the network reported by Collins and colleagues [43], using their reported threshold; PICO E-0 and E-2 networks, which are PPI sets from Hart and coworkers [29]; and MIPS, including all PPIs in physical complexes reported by Hart and coworkers [29], derived from the work reported by Guldener and colleagues [44]. In all cases, self interactions were removed.

For each phenotype, the predictability was evaluated by generating a ROC curve based upon the gene ranking and calculating the AUC. The ROC curve indicates the relative rate of true and false positive predictions as a function of the score *S*_*i*_, plotting the true positive rate (TP/[TP + FN]) versus false positive rate (FP/[FP + TN]). In calculating *S*_*i*_, self-self links were not permitted, and each gene in the seed set was withheld in turn from the seed set for evaluation (leave-one-out cross-validation). TP was defined (for a specific threshold) as the number of genes from the seed set ranked above a given *S*_*i*_. FP was defined as the number of genes above the threshold but not in the seed set. FN was defined as the number of seed genes ranked below the threshold. Finally, TN was defined as the number of nonseed genes ranked below the threshold.

The AUC ranges from 0 to 1, with 0.5 indicating random performance and 1.0 indicating perfect classification. Note that the AUC is calculated using only seed genes represented in the network (the network is not penalized for partial coverage of the seed set), allowing the predictive capacity of networks of differing sizes to be compared. For the purposes of calculating a ROC curve, all genes not linked to the phenotype seed set were treated as being of the same rank. Note that none of the phenotypes have been tested for all genes (most tested only non-essential genes). Because of ambiguities in the reporting of genes tested, ROC curves for the set of 100 phenotypes were calculated over the entire set of yeast genes in the network being tested (5,483 genes for the functional network). Thus, the measures of predictability (AUC) are likely to be underestimates, because all untested genes are considered false positives.

As an alternative test for functional enrichment, we used ArrayPlex [71] to calculate the hypergeometric probability of the enrichment for each GO annotation within a given gene set.

### Prediction of human disease gene sets

For the test of human disease gene prediction, we collected sets of yeast genes whose human orthologs were linked to the same OMIM disease [51]. Human disease phenotypes from OMIM were collapsed into major categories (variants of each disease were collapsed into a single category, such as collapsing 'Cataract, polymorphic and lamellar' and 'Cataract, crystalline aculeiform' into a single category of cataract defects). Each human disease gene was mapped to one of 2,151 human-yeast orthology groups using Inparanoid [72], and seed sets of yeast genes linked to the same disease were selected such that at least four of the yeast genes were present in YeastNet. Calculation of predictability and measurement of AUC was performed as for yeast phenotypes, considering linkages in YeastNet between human-yeast orthology groups rather than between individual yeast genes.

### Generation of random phenotype sets

In order to estimate the random distribution of AUC scores for literature phenotypes, sets of genes of the same sizes as the real phenotype seed sets were drawn from the complete set of yeast genes and tested for predictability, using as the background set of genes those designated by SGD as 'verified' or 'uncharacterized' (not dubious or pseudogenes; as of 29 January 2007). For SCMD morphology phenotypes [50], 1,000 sets of 40 genes were drawn randomly from the complete set of genes analyzed by SCMD, and then tested for predictability in order to generate the null expectation for the AUC distribution. For human disease phenotypes, random gene sets were generated for comparison by randomly drawing from the set of network annotated human-yeast orthologs such that the set size distribution of the random sets matched the size distribution of the actual OMIM disease seed sets.

### Yeast strains, media, and growth

For predicting elongation mutants, we employed a seed set of 77 nonessential genes identified by Giaever and coworkers [4] as 'Elongate 3' in a screen of the homozygous diploid yeast deletion collection. Using GBA with this seed set, we predicted additional genes likely to give rise to elongated cells, and selected for assay the 35 top-ranked essential genes with strains available in the tetracycline downregulatable library of yeast strains [47]. A negative set of 17 strains from the same library was randomly selected from those genes not linked to any of the known elongated genes. The corresponding strains were obtained from Open Biosystems (Huntsville, Alabama, USA). Each strain was grown to saturation at 30°C in rich media (yeast extract/peptone/dextrose (YPD), inoculated into fresh YPD with 10 ng/ml doxycycline, grown 16 hours, and imaged [47] to evaluate cell morphology. Two biologists evaluated the images for each strain (with strain names hidden) for elongated cell morphologies using a simple qualitative scoring scheme (0 to 2), assigning a final score to each strain as the sum of the independent evaluations. Strains scoring more than 2 were selected as elongated, which minimized false positives, yet recovered NUT2, previously reported to be elongated [47].

## Abbreviations

AUC, area under the curve; CV, coefficient of variance; DIP, Database of Interacting Proteins; FN, false negative; FP, false positive; GBA, guilt-by-association; GO, Gene Ontology; MIPS, Munich Information Center for Protein Sequences; OMIM, Online Mendelian Inheritance in Man; PICO, Probabilistic Integrated Co-complex; ROC, receiver operating characteristic; SCMD, *S. cerevisiae *Morphology Database; SGD, *Saccharomyces *Genome Database; TN, true negative; TP, true positive.

## Authors' contributions

KLM, IL, and EMM conceived of the research. KLM performed the experiments. KLM and EMM wrote the paper.

## Additional data files

The following additional data are available with the online version of this paper. Additional data file 1 shows the complete set of 100 phenotypic seed gene sets.
